# Molecular pathogenesis and treatment of cavernous nerve injury-induced erectile dysfunction: A narrative review

**DOI:** 10.3389/fphys.2022.1029650

**Published:** 2022-10-06

**Authors:** Guoda Song, Peng Hu, Jingyu Song, Jihong Liu, Yajun Ruan

**Affiliations:** ^1^ Department of Urology, Tongji Hospital, Tongji Medical College, Huazhong University of Science and Technology, Wuhan, Hubei, China; ^2^ Second Clinical College, Tongji Medical College, Huazhong University of Science and Technology, Wuhan, Hubei, China; ^3^ Department of Plastic Surgery, Tongji Hospital, Tongji Medical College, Huazhong University of Science and Technology, Wuhan, Hubei, China

**Keywords:** erectile dysfunction, cavernous nerve injury, signaling pathway, pathogenesis, nerve regeneration

## Abstract

**Introduction:** Erectile dysfunction (ED) is a common complication after radical prostatectomy (RP), and it seriously affects the quality of life in patients and their partners. The primary trigger of postoperative ED is surgical injury to the cavernous nerves that control penile erection and run along the anterolateral aspect of the prostate. Despite the introduction and ongoing innovation of nerve-sparing techniques, a significant number of patients still suffer from moderate cavernous nerve injury (CNI), which is thought to be transient and reversible. Therefore, early postoperative penile rehabilitation therapy may salvage patients’ erectile function by promoting cavernous nerve regeneration and preventing penile structural alterations.

**Aims:** To present a comprehensive overview of the current molecular pathogenesis of CNI-induced ED, as well as novel therapeutic strategies and their potential mechanisms.

**Methods:** A literature search was performed using PubMed. Search terms included *erectile dysfunction*, *cavernous nerve injury*, *pathogenesis*, *pathway*, and *treatment*.

**Results:** The NOS/NO pathway, oxidative stress-related pathway, RhoA/ROCK pathway, transforming growth factor-β (TGF-β), sonic hedgehog (Shh), and hydrogen sulfide (H_2_S) are involved in the molecular pathogenesis of CNI-induced ED. Multiple neurotrophins, including brain-derived nerve growth factor (BDNF), glial cell line-derived neurotrophic factor (GDNF), and neurturin (NTN), were found to promote cavernous nerve regeneration. Emerging therapeutic approaches can be roughly summarized into four categories, namely small molecule and drug, stem cell-based therapy (SCT), micro-energy therapy and platelet-rich plasma (PRP) therapy.

**Conclusion:** These pathways collectively lead to the irreversible damage to the penile structure after CNI. The combined early rehabilitation strategies of promoting upstream nerve regeneration and recovering abnormal molecular signals of downstream penis are presumed to save patients’ erectile function after RP. In future studies, the cross-talk between these molecular pathways needs to be further clarified, and the questions of how denervation injury induces the molecular alterations in the penis also need to be addressed.

## 1 Introduction

Neurogenic erectile dysfunction is caused by insufficient nerve signal transmission to the corpus cavernosum. The main etiology of neurogenic ED can be classified as central lesions (e.g., multiple sclerosis, Parkinson’s disease, spinal cord injury, *etc.*) and peripheral lesions (e.g., cavernous nerve injury caused by pelvic surgery or pelvic fracture) (Thomas and Konstantinidis, 2021), among which, cavernous nerve injury (CNI) caused by radical pelvic surgery (especially radical prostatectomy) as the main cause of iatrogenic ED has been recognized as a point of concern for clinicians. Several high-quality studies have exhibited that up to 80% of patients develop ED after radical prostatectomy (RP) (Emanu et al., 2016). Even with meticulous nerve-sparing radical prostatectomy (NSRP) performed by skilled surgeons, it is still inevitable that many patients experience declines in erectile function after surgery. A study by Tal and colleagues in 2009 showed that the incidence of venous leakage at 6 months after bilateral NSRP was 7%, and at 18 months, the recovery rate of erectile function without drug assistance was 49% (Tal et al., 2009). With continuous innovations in prostatic procedures and ever-growing knowledge of prostatic anatomy, especially the application of robotic-assisted technology, which enhances the accuracy of surgery, it is possible to improve the preservation of periprostatic neurovascular bundles (NVBs). Nonetheless, several meta-analyses have suggested that whether robotic procedures can improve postoperative erectile function is still debatable (Ficarra et al., 2012; Du et al., 2018; Wang et al., 2018). Currently, phosphodiesterase type 5 inhibitor (PDE5I) therapy is the first-line treatment for erectile dysfunction. A meta-analysis performed by Chen et al. (2015) presented that the overall effective rate of sildenafil 50 mg is up to 47% against placebo. However, PDE5I therapy is not quite satisfactory for the recovery of erectile function after RP. Meta-analyses performed by Limoncin et al. (2017) and Liu et al. (2017) showed that PDE5Is have no significant effect on the amelioration of postoperative spontaneous (no drug-assisted) erectile function and can merely improve the rate of drug-assisted EF recovery by 10% or more as compared to placebo. Due to the fact that there is no surgical technique to completely avoid cavernous nerve injury and the curative effect of penile rehabilitation therapy is still uncertain, it has become an important issue to clarify the molecular pathogenesis of CNI-related ED and to explore new treatments for the restoration of postoperative erectile function. This review presents an overview of the research progress in the molecular pathogenesis, molecular signaling pathways, and novel therapies of CNI-related ED.

## 2 Neuropathology and animal models of cavernous nerve injury

According to the Seddon classification, peripheral nerve injury is stratified into three degrees: neurapraxia, axonotmesis, and neurotmesis, whose severity increases in turn, as well as recovery time (Kaya and Sarikcioglu, 2015). Neurapraxia is primarily a demyelinating injury, which means the connections of axons remain mostly unimpaired and Wallerian degeneration hardly occurs. Thus, the injury site can be remyelinated and repaired by Schwann cells, and the conduction of the nerve will soon be well restored. In terms of axonotmesis, due to the interruption of axons, Wallerian degeneration occurs on the segment distal from the site of injury, and only the surrounding supporting connective tissues remain partially or fully retained. Consequently, the recovery time of nerve conduction is prolonged, depending on the capacity of axonal regeneration and the integrity of surrounding supporting tissues after injury (Robinson, 2018). The cavernous nerve which originates from the major pelvic ganglion (MPG), contains both parasympathetic (nitrergic) nerves and sympathetic nerves, mediating the relaxation and contraction of cavernous smooth muscle respectively, together regulating the erectile function of penile tissue (Dean and Lue, 2005). Hsieh et al. (2003) have proven that the ratio of parasympathetic nerves to sympathetic nerves decreased after CNI, and the sympathetic nerve components showed a stronger ability to regenerate after injury. Hence, they proposed a hypothesis that the imbalance of these two nerve components may be responsible for RP-related ED. NSRP strives to preserve the continuity of the cavernous nerve as much as possible, but during the operation, the neurovascular bundles are inevitably affected by traction, crushing, and/or thermal injury, resulting in neurapraxia or mild axonotmesis. Although the nerve remains intact in appearance, its transmission will be temporarily blocked, which is believed to cause structural changes in penile tissue (Fode et al., 2013). During this transmission-blocking period, the penile tissue is in a persistent state of ischemia and hypoxia, which may lead to smooth muscle apoptosis and tissue fibrosis, which subsequently leads to venous leakage. Thus, it is convincing that this kind of structural changes may be an essential component for long-term ED after NSRP (Fode et al., 2013). However, the molecular mechanism behind this pathophysiological process has not been elucidated, leading to the inaccurate effect of current postoperative penile rehabilitation.

At present, the animal model of CNI takes rat as the main carrier, a recent consensus statement (Weyne et al., 2020) recommended several standardized guidelines for the construction of CNI models, including but not limited to: I, bilateral rather than unilateral CNI should be used as the standard model; II, important parameters such as injury mode (e.g., crush, transection, heat. Crush injury is the most common method to simulate nerve injury caused by NSRP), used instrument and injury duration should be recorded in detail; III, priority should be given to intracavernous pressure (ICP) rather than corpus spongiosum pressure (CSP) as an index to evaluate erectile function. In conclusion, the cavernous nerve crush rat model is recommended for simulating the condition of moderate CNI in post-RP patients.

## 3 Molecular pathogenesis of cavernous nerve injury-erectile dysfunction

### 3.1 Nitric oxide synthase/nitric oxide pathway

Nitric oxide (NO), a key molecule in mediating penile erection, is catalyzed from L-arginine by nitric oxide synthase (NOS). The NOS/NO signaling pathway, which is currently the most important and comprehensive understanding pathway of physiological erection, also plays a crucial role in the pathogenesis of CNI-ED. Once sexual signals are transmitted to the corpus cavernosum *via* cavernous nerve, NO is generated in nitrergic nerve terminals and endothelial cells of the cavernosa under the activation of nNOS and eNOS, respectively. Synthetic NO then diffuses into corpora cavernosum smooth muscle cells (CCSMCs) to bind and stimulate soluble guanylate cyclase (sGC), which converts GTP into the second messenger cGMP. cGMP activates cGMP-specific protein kinase (PKG), which phosphorylates and regulates a variety of ion channels: I, it inhibits the activity of transmembrane L-type calcium channels, consequently preventing extracellular calcium influx; II, it promotes the transfer of cytoplasmic calcium into the endoplasmic reticulum, together resulting in a decline in cytoplasmic calcium concentration; III, it inhibits calcium-activated chloride channels (CaCCs), causing a reduction of in chloride outflow. IV, it activates transmembrane large conductance Ca^2+^-activated K^+^ channel (BKCa) and ATP-dependent K^+^ channel (KATP), promoting intracellular potassium outflow and generating intracellular hyperpolarization potential, resulting in the suppression of calcium channel activation and further reduction of cytoplasmic Ca^2+^ concentration. The reduction in cytoplasmic Ca^2+^ ultimately induces dephosphorylation of myosin light chain (MLC), causing relaxation of CCSMCs, vasocongestion of penis, and erection. PDE type 5 (PDE5), which catalyzes and deactivates cGMP into 5′-GMP, is the most active of the thirteen PDEs found in cavernosal tissue and its inhibitor is currently the most effective drug for the treatment of ED (Kuthe et al., 2001).

It has been demonstrated that the expression and activity of nNOS protein decrease in the MPG and penis of CNI rats, resulting in the decline of nerve-derived NO, which in turn affects the relaxation of corpus cavernosum smooth muscle and erectile function (Karakus et al., 2017). The phosphorylation of nNOS is one of the main mechanisms for regulating the bioactivity of nNOS, which can be mediated by several protein kinases: protein kinase A (PKA), protein kinase B [Akt (or PKB)], AMP-activated protein kinase, and Ca2+/calmodulin-dependent protein kinase II (Murphy et al., 2009; Song et al., 2012). There is evidence that phosphorylation of the nNOS Ser^1412^ site activates nNOS, while the phosphorylation of Ser^847^ inhibits its activity (Rameau et al., 2007). Hurt et al. have revealed the important role of PKA-mediated Ser^1412^ phosphorylation of nNOS in physiological erection (Hurt et al., 2012). Their subsequent study further indicated the favorable regulatory effect of PKA activator colforsin on nNOS after CNI (Karakus et al., 2017). The results showed that the phosphorylation of Ser^1412^ and Ser^847^ of nNOS in the MPG and penis were both significantly up-regulated after CNI, suggesting that hyperphosphorylation of Ser^1412^ may conversely lead to inactivation of nNOS, which seems inconsistent with previous study. Furthermore, the level of nNOS uncoupling and protein inhibitor of nNOS (PIN) binding to nNOS were also upregulated. nNOS has the catalytic activity to generate NO when it is in the dimer state. In contrast, when it is uncoupled as a monomer, its activity for the formation of NO is lost. Instead, the ability to catalyze the production of reactive oxygen species (ROS) is significantly elevated, which is supported by the upregulation of H_2_O_2_ and total ROS in the penis. It has been elucidated that ROS not only contribute to the reduction of NO bioavailability (Jones et al., 2002), but also lead to structural impairment of penile tissue (Lagoda et al., 2007). The main causes of nNOS uncoupling were considered to be the phosphorylation of Ser^847^ of nNOS and the increase of PIN. Moreover, the study also identified that NADPH oxidase subunit gp^91^phoxin was upregulated in the MPG after CNI, indicating that NADPH oxidase may be another source of oxidative stress in the MPG. In summary, the authors proposed a hypothesis that the inactivation of nNOS and the increase of oxidative stress collectively leads to the occurrence of neurogenic ED. The PKA activator colforsin reversed these molecular changes, namely reduced the hyperphosphorylation of Ser^1412^ and Ser^847^, prevented the uncoupling of nNOS and diminished the level of oxidative stress, thus improving erectile function. However, the potential mechanism of Colforsin reversing these molecular changes remains to be further investigated.

In addition, nNOS could indirectly enhance the activity of eNOS by increasing penile blood flow and shear stress, whose molecular mechanism is to activate the phosphatidylinositol 3-kinase (PI3K)/Akt pathway and consequently phosphorylate Ser^1177^ of eNOS (Hurt et al., 2002; Hurt et al., 2012). Therefore, it is reasonable to assume that the downregulation of expression and activity of nNOS after CNI leads to a corresponding reduction of eNOS-dependent NO synthesis, together resulting in persistent flaccidity and low oxygen supply in the penis. Some scholars presume that long-term hypoxia may lead to apoptosis and fibrosis of penile smooth muscle cells, damaging the vital mechanism of venous occlusion (Fode et al., 2013). It has been observed that hypoxia could induce the expression of transforming growth factor-β1 (TGF-β1) and inhibit the synthesis of prostaglandin E in cultured CCSMCs (Moreland, 1998; Leungwattanakij et al., 2003). TGF-β1 is recognized to induce collagen synthesis, while prostaglandin E may inhibit it. Nevertheless, the specific molecular mechanism of penile structural changes caused by long-term hypoxia remains to be further clarified.

A recent study (Musicki et al., 2018) also revealed that the S-nitrosylation modification of eNOS and its downstream signaling molecule sGC in the penis mediates the occurrence of CNI-ED. S-nitrosylation refers to the covalent modification of protein cysteine residues by NO to form an S-nitrosothiol (SNO) (Lima et al., 2010), and the effect of NO on protein S-nitrosylation is independent of the cGMP/PKG pathway. S-nitrosylation is involved in the post-translational modification of many proteins, including NOS and sGC itself in the classical NOS/NO pathway. This study indicated that the S-nitrosylation modification of eNOS and sGC inhibits their catalytic activity, while N-acetyl-cysteine (NAC, a de-S-nitrosylation reagent) can preserve the effect of the NO/cGMP signaling pathway and protect erectile function (Musicki et al., 2018).

### 3.2 Oxidative stress-related pathway

Oxidative stress is a state of imbalance between oxidation and antioxidation, which is considered to be an important factor leading to aging and disease. Wang et al. (2019); (Wang et al., 2015); found that the levels of glutathione peroxidase (GPX) and 3-nitrotyrosine (markers of oxidative stress) in the penis of CNI rats were significantly increased, and suggested that oxidative stress may be an important mechanism of CNI-ED. In addition, oxidative stress has also been shown to be involved in other types of ED, such as hypertension-related, diabetic, and radiation-induced ED (Jin et al., 2008; Suresh and Prakash, 2011; Kimura et al., 2012). Free radicals in the human body include ROS and reactive nitrogen species (RNS). Although the source of free radicals in penis after CNI has not been fully understood, according to the evidence of other types of ED, it is reasonable to speculate that NADPH oxidase (NOX) is activated after CNI and plays an important role in upregulating the level of oxidative stress (Jin et al., 2008; Kimura et al., 2012). NOX catalyzes NADPH to form superoxide anion (O^2-^), which reacts with NO to form peroxynitrite (ONOO-), thus reducing the content of NO (Agarwal et al., 2006). In addition, uncoupled nNOS has been elucidated to be another source of ROS after CNI (Karakus et al., 2017). Besides reducing the content of NO, oxidative stress may also induce corpus cavernosum fibrosis, promote apoptosis of smooth muscle cells, and lead to endothelial dysfunction (Kimura et al., 2012). Furthermore, it has been shown that oxidative stress can also inhibit cavernous nerve regeneration and induce apoptosis of MPG neurons after CNI (Zhao et al., 2016). Therefore, reducing the level of oxidative stress is indeed an alternative and efficient therapy to improve erectile function after RP.

### 3.3 Transforming growth factor-β pathway

Many studies have shown that the expression of TGF-β increases significantly after CNI, which is associated with cavernous fibrosis (Leungwattanakij et al., 2003; Hu et al., 2004; Shin et al., 2011). After binding to type II receptor (TGF-βRII), TGF-β recruits the type I receptor (TGF-βRI) and bridges it with the type II receptor to form a receptor complex. There is a highly conserved near-membrane domain (also known as the GS domain) rich in Gly and Ser on TGF-βRI. Several Ser and Thr residues in the GS domain are phosphorylated by type II receptors to activate TGF-βRI. Activated TGF-βRI then recruits and phosphorylates the downstream signaling molecules SMAD family members SMAD2 and/or SMAD3, which subsequently combine with SMAD4 to form a heterotrimeric complex. The SMAD complex is subsequently transported into the nucleus to regulate the transcription of TGF-β target genes, thus inducing collagen formation and fibrosis-related changes (Roberts and Derynck, 2001; Schmierer and Hill, 2007; Shin et al., 2011). In addition, SMAD7 has also been proven to be involved in the above pathway, which inhibits the phosphorylation of SMAD2 and SMAD3 mediated by TGF-βRI, thereby inhibiting downstream signal transmission. Song et al. (2014) used adenovirus to transfect the SMAD7 gene into the corpus cavernosum of CNI mice and successfully restored erectile function *via* hindering penile fibrosis, inhibiting endothelial cell apoptosis and enhancing the phosphorylation of Ser^1177^ of eNOS. Previous studies have elucidated that the TGF-β/SMAD pathway mediates apoptosis of vascular endothelial cells (Lu et al., 2009) and smooth muscle cells (Redondo et al., 2005) in other organs, yet the role of this pathway in penile tissue apoptosis after CNI remains uncertain. Moreover, a study by Miyajima et al. (2000) demonstrated that there is a correlation between TGF-β and the expression and activity of NOS in the kidney with unilateral ureteral obstruction, so whether TGF-β after CNI affects the activity of eNOS and its molecular mechanism are also worthy of further investigation.

TGF-β may also induce CNI-related ED *via* non-SMAD signaling pathways, such as the RhoA/ROCK, RAS/MEK/ERK, and PI3K pathways (Derynck and Zhang, 2003). Hannan et al. (2014) found that the expression of histone deacetylase (HDAC) family members HDAC3 and HDAC4 is up-regulated in the penis of CNI rats, and is involved in the induction of penile fibrosis. Intraperitoneal injection of the HDAC inhibitor valproic acid (VPA) reverses fibrosis and improves erectile function. HDAC regulates gene transcription *via* catalyzing the deacetylation of acetyl-L-lysine side chains in histones (Lombardi et al., 2011). Additionally, many transcriptional factors and signaling proteins and other non-histone proteins also act as targets of HDAC to regulate a variety of biological functions (Glozak et al., 2005). There is evidence (Barter et al., 2010) that HDAC is necessary for TGF-β to activate ERK and PI3K pathways and to induce subsequent expression of fibrogenic genes. The above studies not only suggest that HDAC is an effective molecular target for reversing penile fibrosis induced by TGF-β after CNI, but also imply that the SMAD-independent pathways of TGF-β may be involved in the profibrotic cascade. Furthermore, the studies by Cho et al. (2011); (Song et al., 2015) indicated that the sphingosine-1-phosphate (S1P) and RhoA/ROCK1 signals may mediate cavernous fibrosis *via* cooperating with TGF-β after CNI, which also supports the above hypothesis. In conclusion, it is significant to clarify the driving role of these TGF-β atypical pathways in CNI-ED, as relevant studies may reveal the complex cross-talk between these pathways and provide novel molecular targets for the selection of specific drugs.

### 3.4 RhoA/ROCK pathway

Rho, a small monomer of the G protein Ras superfamily, is a 20–30 KD GTP binding protein with GTP enzyme activity. Rho-associated kinase (ROCK) belongs to the serine/threonine kinase family and includes two isoforms, ROCK1 and ROCK2. The structure of ROCK consists of three parts: an N-terminal serine/threonine kinase domain, a pleckstrin homology domain at the carboxyl terminus, and a helix domain containing the Rho-binding domain (Loirand, 2015). RhoA/ROCK exists in many tissues throughout the human body and participates in regulating a variety of physiological functions, including but not limited to, cell contraction, migration, proliferation, and adhesion. In Chitaley et al. (2001) detected the expression of endogenous ROCK in corpus cavernosum for the first time and revealed the effect of RhoA/ROCK pathway on penile erection. In the process of mediating the contraction of CCSMCs, ligands such as endothelin-1 (ET-1), angiotensin II (Ang II) and norepinephrine are released from endothelial cells and cavernous nerve terminals above all. Subsequently, ligands bind to the G protein-coupled receptor (GPCR) of smooth muscle cells to activate guanine exchange factor (GEF), which converts inactive RhoA-GDP into active RhoA-GTP. RhoA-GTP then detaches from the RhoA-GDP dissociation inhibitor and translocates to the cell membrane, where it binds to ROCK and leads to the consequent autophosphorylation and activation of ROCK. Activated ROCK then facilitates the phosphorylation of myosin light chain phosphatase (MLCP). Unphosphorylated MLCP can dephosphorylate myosin light chain (MLC) and promote the release of myosin from actin and the relaxation of smooth muscle. Phosphorylated MLCP acts conversely, resulting in contraction of smooth muscle (Sopko et al., 2014). Previous studies (Gratzke et al., 2010; Sopko et al., 2014) suggested that ROCK2 is the primary isoform up-regulating in the penis of CNI-ED rats, while ROCK1 is overexpressed in diabetic ED model, which yet remains uncertain and requires further elucidation.

In addition to mediating the contraction of CCSMCs, it has also been revealed that the activation of the RhoA/ROCK pathway in penile tissue of CNI rats leads to the induction of corporal apoptosis and fibrosis (Cho et al., 2011; Hannan et al., 2013; Cho et al., 2015). The TGF-β/S1P/RhoA/ROCK1/LIMK2/cofilin pathway has been indicated to mediate penile fibrosis after CNI (Cho et al., 2011; Song et al., 2015), while the RhoA/ROCK/Akt/Bad/Bax/caspase-3 pathway may be involved in cavernous smooth muscle apoptosis (Cho et al., 2015). In addition, Hannan et al. (2016) showed that RhoA/ROCK is also activated in the MPG after CNI and induces the caspase-3-dependent apoptosis of nitrergic neurons. The application of ROCK inhibitor Y-27632 promotes the outgrowth of axons of cultured MPGs *in vitro*.

The RhoA/ROCK pathway regulates the tension of penile smooth muscle in an NO-independent manner, but several studies have suggested that there may be a wide correlation between the RhoA/ROCK pathway and NO pathway. Up-regulation of RhoA/ROCK can inhibit the expression and activity of eNOS in penis (Hannan et al., 2013). The underlying mechanism of low expression of eNOS may be attributed to the fact that RhoA/ROCK reduces the stability of NOS3 mRNA, which encodes eNOS (Laufs and Liao, 1998). Meanwhile, ROCK reduces the activity of eNOS by directly phosphorylating Thr^495^ of eNOS or indirectly phosphorylating and deactivating the upstream regulatory molecule PKB of eNOS (Ming et al., 2002; Sugimoto et al., 2007). In addition, intraperitoneal injection of ROCK inhibitor Y-27632 into CNI rats could prevent the uncoupling of nNOS in the MPG, increase the expression and activity of nNOS in the MPG and partially in cavernous tissue (Hannan et al., 2013; Hannan et al., 2016), which may be related to the neuroprotective effect of Y-27632 on the MPG/CN. Moreover, PKG can prevent RhoA-GTP binding to ROCK by phosphorylating it, thus inhibiting the activity of ROCK (Sauzeau et al., 2000).

### 3.5 Sonic hedgehog pathway

Sonic hedgehog (Shh) protein is a secretory protein that belongs to the Hedgehog (Hh) family, along with Indian Hedgehog (Ihh) and Desert Hedgehog (Dhh). The evolutionarily conserved Hh pathway is required for proper embryonic development and plays an important role in adult tissue maintenance, renewal, and regeneration (Beachy et al., 2010). There is evidence showing that the expression of Shh is essential for penile embryonic development, postnatal differentiation, and maintenance of adult penile tissue integrity (Podlasek et al., 2005). Shh protein is mostly abundant in the smooth muscle of cavernous sinus, as well as the MPG and cavernous nerve that innervate the corpus cavernosum (Podlasek, 2009). The Shh pathway has been thoroughly explored in embryonic development and some other organs, but there are few reports on it in adult penis and MPG.

#### 3.5.1 Typical sonic hedgehog pathway

The Patched 1 (PTCH1)-Smoothened (SMO)-suppressor of fused (SUFU)-GLI axis is the core component of the typical signaling pathway of Shh (Ingham et al., 2011). The Shh protein initiates signal transduction by binding to the typical receptors PTCH1 and coreceptors growth arrest-specific 1 (GAS1), CAM-related/downregulated by oncogenes (CDO), brother of CDO (BOC) and low-density lipoprotein receptor-related protein 2 (LRP2). PTCH1 is concentrated in and around the primary cilia. When Shh does not bind to PTCH1, PTCH1 inhibits the activity of SMO. Without activated SMO, the GLI proteins (GLI2 and GLI3) that bind to SUFU are first phosphorylated by PKA, and then further phosphorylated by glycogen synthase kinase 3 (GSK3) and casein kinase 1 (CK1). Phosphorylated GLIs are hydrolyzed and cleaved to generate the transcriptional repressor forms GLIRs. Once Shh binds to PTCH1, PTCH1 leaves the primary cilia, releasing the inhibition on SMO. SMO is subsequently phosphorylated by G protein-coupled receptor kinase 2 (GPRK2) and CK1, enters the primary cilia together with β-arrestin and the microtubule motor KIF3A, and enriches in the cilia with the assistance of Ellis-van Creveld syndrome protein (EVC) and EVC2. Enriched SMO induces the separation of GLI from SUFU, allowing GLI to bypass the proteolytic processing and transport into the nucleus in its full-length activated form, which acts as a transcriptional activator to regulate the transcription of target genes (Podlasek, 2009; Ingham et al., 2011; Briscoe and Therond, 2013). The target genes of Shh pathway include vascular endothelial growth factor (VEGF), NOS, BMP-4 and Hoxd-13 (Podlasek et al., 2005; Podlasek et al., 2007; Bond et al., 2013a), all of which are linked to the development of ED. GLI, as a major transcriptional regulator in the Shh pathway, has the capacity to control transcription in both directions. In the absence of Shh signaling, GLI3 acts as the primary transcriptional suppressor. Once Shh ligand is present, GLI2 acts as the main activator, triggering the expression of GLI1 and other Shh target genes. GLI1, as another powerful transcriptional activator, further enhances the expression of target genes (Hui and Angers, 2011).

#### 3.5.2 Atypical sonic hedgehog pathway

The term “atypical Shh pathway” is not well defined. At present, it may be classified into two categories: one is mediated by PTCH1, but independent of its inhibition on SMO. PTCH1 has been shown to trigger apoptosis through the DRAL-caspase-9 complex in the absence of Shh (Guerrero and Ruizi Altaba, 2003; Thibert et al., 2003; Mille et al., 2009). The other is mediated by SMO but independent of GLI signaling. For example, Shh induces axonal growth through SMO-mediated activation of Src family kinases (SFKs) (Yam et al., 2009); Shh promotes fibroblast migration by activating small Rho GTP enzymes Rac1 and RhoA through SMO (Polizio et al., 2011). In addition, the GLI-mediated pathway independent of SMO regulation is also considered as one of the atypical Shh pathways (Teperino et al., 2014).

Podlasek et al. (2007); (Bond et al., 2008) observed that the Shh protein in penile tissue of CNI Sprague Dawley rats was significantly decreased, resulting in abundant apoptosis of CCMSCs. Intracavernous injection of Affi-Gel beads containing Shh inhibitor into normal rats increased the apoptosis of CCMSCs by 12-fold as compared with the control group. In contrast, intracavernous injection of Shh protein could prevent apoptosis of corpus cavernosum cells. Thereafter, more research was conducted to better understand the involvement of the Shh pathway in MPG/CN. The results showed that Shh protein is vital to maintain the integrity of the CN and that inhibition of Shh signaling in the MPG leads to CN demyelination and axonal degeneration. Conversely, the application of Shh protein to the damaged CN nerve can promote CN regeneration, inhibit penile cell apoptosis and eventually improve erectile function (Angeloni et al., 2011; Dobbs et al., 2018). The molecular mechanism by which Shh promotes CN regeneration is not entirely understood, although part of it is related to the upregulation of brain-derived neurotrophic factor (BDNF), which has been determined to have a neuroprotective effect on nNOS positive neurons (Bond et al., 2013b). Furthermore, it has also been proven that BDNF promotes axonal growth by activating the JAK/STAT pathway after CN injury (Lin et al., 2010). Shh protein is abundantly expressed in MPG neurons and may have a paracrine effect on satellite glial cells surrounding the MPG (Angeloni et al., 2013). The communication between neurons and glial cells carried out by the Shh signaling pathway may play an important role in neuroprotection and regeneration, and further studies should be conducted to determine whether Shh directly mediates above communication or indirectly mediates its downstream target molecules. Shh is also abundant in Schwann cells of CN, which seems to be necessary for maintaining the integrity of CN and CN regeneration (Bond et al., 2008; Angeloni et al., 2011). Moreover, regarding the mechanism of MPG/CN regulating Shh protein in the penis, the team (Bond et al., 2008; Angeloni et al., 2009) concluded that Shh cannot be transported anterograde from the MPG to the corpus cavernosum through the CN; CN impulses affect the expression of Shh protein in the penis; and some nutritional factors produced in the MPG are delivered by the CN to the corpus cavernosum to control its internal Shh signal. Hedgehog-interacting protein, one of the target proteins of the Shh pathway, has been shown to play such a role, as well as maintain the integrity of the CN.

A recent study (Choe et al., 2016) has shown that the Shh pathway is also implicated in the fibrosis of cavernous tissue following CN injury, and the decrease of Shh protein in the penis leads to a rise in collagen. There is evidence of a relationship between Shh pathway and TGF-β pathway in other diseases (Hu et al., 2015). Nevertheless, the specific mechanism by which Shh promotes fibrosis in penile tissue remains to be further elucidated. In addition, a study (Dobbs et al., 2019) further showed that the Shh pathway can induce the expression of ROCK1 in MPG/CN, suggesting that there may be cross-talk between the Shh pathway and the Rho/ROCK pathway in the pathogenesis of CNI-related ED.

### 3.6 Endogenous hydrogen sulfide pathway

Endogenous hydrogen sulfide (H_2_S) is produced from L-type cysteine (L-Cys) or homocysteine under the catalysis of cystathionine-β-synthase (CBS), cystathionine-γ-lyase (CSE) or 3-mercaptopyruvate sulfurtransferase (3-MST). As another gas transmitter, H_2_S may play a regulatory role similar to that of NO. Srilatha et al. (2006) first discovered the crucial role of endogenous H_2_S in penile erectile function in 2006, subsequently, Roberta et al. (D’Emmanuele Di Villa Bianca et al., 2009) confirmed that both cavernous nerve and cavernous smooth muscle cells express it. H_2_S in cavernous smooth muscle cells can activate BKCa and KATP ion channels, produce intracellular hyperpolarization potential and promote relaxation, along with its ability to inhibit the expression and activity of NADPH oxidase and reduce the level of oxidative stress. The adenylyl cyclase (AC)/cAMP/PKA cascade may mediate the above H_2_S-induced molecular changes. Meanwhile, H_2_S can directly inhibit the activity of PDE5 and mitigate the degradation of cGMP (Liaw et al., 2011; D’Emmanuele Di Villa Bianca et al., 2011). In addition, H_2_S may also act as an important endogenous regulator of cell proliferation and apoptosis (Kanagy et al., 2017). Although abundant research has revealed the mechanism of H_2_S in penile erection, little is known about the role of H_2_S in the pathogenesis of neurogenic ED. Recently, Zeng et al. (Qinyu et al., 2021) have revealed for the first time that the decrease of H_2_S concentration in penile tissue is related to the occurrence of BCNI-ED, and exogenous H_2_S inhibits the phenotypic transformation of CCSMCs and improves the erectile function of BCNI rats by inhibiting the RhoA/ROCK1 pathway and consequently affecting its downstream factors CDK2, Cyclin E1 and P27^kip1^. The concept of phenotypic transformation of CCSMCs originated from that of vascular smooth muscle cells (VSMCs). It is believed that during the development of vascular pathologies such as intimal hyperplasia, vascular stenosis and atherosclerosis, VSMCs are no longer in a resting state, and the expression of contractile proteins is down-regulated, moreover, the ability to proliferate, migrate and produce extracellular matrix proteins is elevated (Beamish et al., 2010). These processes are defined as the phenotypic transformation of VSMCs, that is, the shift of “contractile” VSMCs to “synthetic” or “proliferative” fibroblast-like VSMCs. A study by Yang et al. (2014) first demonstrated that there is phenotypic transformation of CCSMCs in CNI rats and is associated with penile fibrosis. Subsequent *in vitro* experiments (Lv et al., 2014; Yan et al., 2017) showed that hypoxia could induce the phenotypic transformation, which may be mediated by the platelet derived growth factor (PDGF)/PDGFR/STAT3 signaling pathway. In addition, PDGF also activates the RhoA/ROCK pathway through PDGF receptor to promote the phenotypic transformation of VSMCs and CCSMCs (Tang et al., 2018; Qinyu et al., 2021). H_2_S, as a new therapeutic molecular target in CNI-ED, has been explored to a limited degree. However, how it inhibits the RhoA/ROCK pathway and whether there are alternative H_2_S-dependent signaling pathways involved in CNI-ED still need further elucidation.

## 4 Neurotrophin-related neuroprotective and neuroregenerative pathways

Accumulating evidence has indicated that the temporary blockade of nerve conduction caused by cavernous nerve injury leads to structural changes in penile erectile tissue, which are often difficult to reverse, resulting in long-term and difficultly recovered erectile dysfunction. Therefore, fundamentally promoting the regeneration of injured nerves, specifically nitrergic nerves, and shortening the duration of penile denervation after RP will bring a tremendous therapeutic effect for the restoration of postoperative erectile function. In addition to the above-mentioned SHH and RhoA/ROCK pathways, which may be involved in the processes of cavernous nerve protection and regeneration, neurotrophins, immunophilins, erythropoietin and neuregulins et al. (Campbell and Burnett, 2017) have also been suggested to be alternative targets. Here, we focus on the review of neurotrophins and their possible pathways.

Neurotrophins are a family of proteins that support the survival, development and normal function of neurons. At present, neurotrophins which have been proved to recover erectile function after CNI mainly comprise brain-derived nerve growth factor (BDNF), glial cell line-derived neurotrophic factor (GDNF), neurturin (NTN), growth differentiation factor-5 (GDF-5) and VEGF (Bella et al., 2009; Campbell and Burnett, 2017). The underlying pathways of above neurotrophins promoting cavernous nerve regeneration are mostly unclear. A study by Bella et al. (2006) has shown that BDNF stimulates axonal growth of rat MPGs cultured *in vitro* mainly through the JAK/STAT pathway, while the MEK/ERK and PI3K/Akt pathways activated by tropomyosin-related kinase B (TrkB) and pan-neurotrophin 75 (p75) receptors may only act as auxiliary pathways. It is currently unclear how BDNF activates the JAK/STAT pathway, and a possible mechanism is indirect activation through Schwann cells. GDNF and NTN both belong to the GDNF family, and are similar in physiological function and downstream signaling pathway. GDNF and NTN bind to their receptor GFR-α and activate the Ret receptor tyrosine kinase, thus initiating downstream intracellular signaling pathways, including the PI3K, MAPK and Src family kinase pathways. Although the signaling pathways of GDNF family in cavernous nerve regeneration have not been completely clarified, Wanigasekara and Keast (2005) have proven that NTN initiates axonal growth of parasympathetic neurons of the MPG through the PI3K pathway and regulates microtubule assembly through the MAPK and Src kinase pathways. More interestingly, compared with parasympathetic neurons, the stimulating effect of NTN on axonal growth of sympathetic neurons in MPG is significantly weaker, although the underlying mechanism is yet unknown, the inconsistent effects on promoting axonal regeneration seem to be more helpful in normalizing the imbalance between sympathetic and parasympathetic innervation after CNI, therefore, are more conducive to the recovery of erectile function. GDF-5 is a member of the bone morphogenetic protein (BMP)/TGF-β superfamily, the possible candidate pathways of which for its neuroprotective effect include Smad and p38 mitogen-activated protein kinase (MAPK) pathways (Bella et al., 2009). In addition to the neuroprotective effect, Fandel et al. (Fandel et al., 2008) found that intracavernous injection of GDF-5 can significantly reduce the level of TGF-β mRNA in the penis after CNI in a dose-dependent manner, which may be attributed to the direct competition between GDF5 and TGF-β for Smad pathway and inhibition of TGF-β-induced self-expression. This additional effect also suggests that NTN may improve erectile function after RP through multiple-site protective effects and increase the possibility of clinical translation. With regard to VEGF, Chen et al. (2005) have demonstrated that intracavernous injection of VEGF could enhance cavernous nerve regeneration, and co-administration with BDNF could further improve the curative effect. Furthermore, another study (Yang et al., 2020) showed that transplantation of adipose stem cells (ADSCs) coexpressing VEGF and GDNF around the MPG could rapidly repair injured cavernous nerves. VEGF not only promotes angiogenesis and vascular permeability, but also has neurotrophic activity and stimulates axonal growth through flk-1 receptors (Zhang et al., 2010). Possible pathways of VEGF-mediated cavernous nerve regeneration include the Ras/Raf, PI3K/Akt or JAK/STAT pathways, yet, require further investigation.

In addition to the above-mentioned neurotrophic factors, some new molecules conducive to cavernous nerve regeneration have been found in recent years, such as galanin, insulin-like growth factor-1 (IGF-1) and LM11A-31 (Weyne et al., 2018; Haney et al., 2019; Yin et al., 2021), which exhibit significant effects on the restoration of postoperative erectile function in rat models, and need more studies to clarify their potential pathways.

**FIGURE 1:**
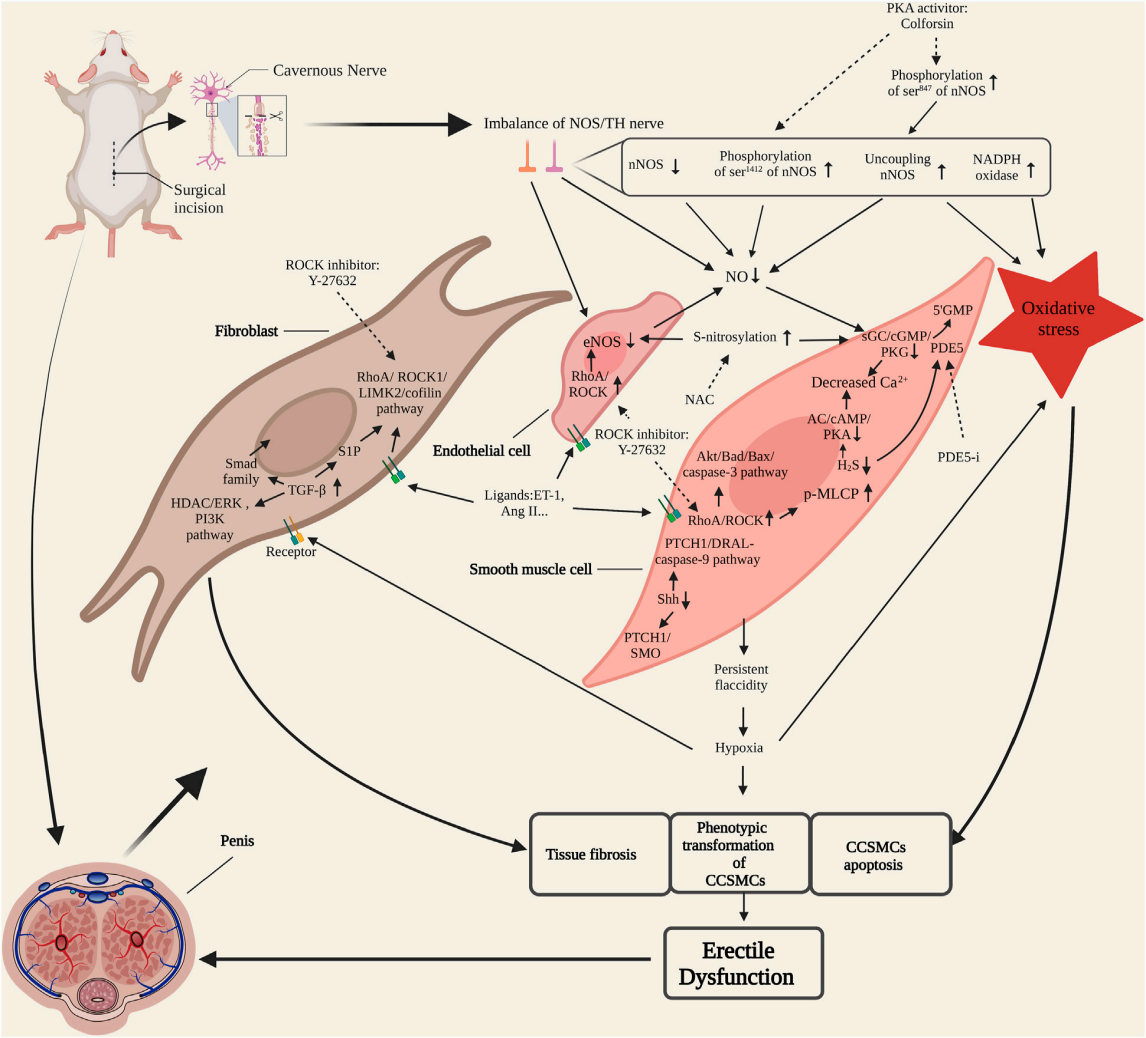
Molecular mechanism of cavernous nerve injury-erectile dysfunction (CNI-ED). NOS/TH nerve, nitrergic and sympathetic nerve. CCSMCs, corpora cavernosum smooth muscle cells; NOS, nitric oxide synthase; PKA, protein kinase A; NO, nitric oxide; sGC, soluble guanylate cyclase; PKG: cGMP-specific protein kinase; PDE5, phosphodiesterase type 5; PDE5-i, phosphodiesterase type 5 inhibitor; AC, adenylate cyclase; NAC, N-acetyl-cysteine; ET-1, endothelin; Ang II, angiotensin II; TGF-β, transforming growth factor-β; S1P, sphingosine-1-phosphate. Dotted arrow represents inhibition.

In conclusion, the molecular mechanism of CNI-ED is showed in Figure 1. After cavernous nerve injury, the penile tissue loses the control of nitrergic nerve. The molecular substance such as NO and H2S secretion from the nerve declines which leads to the persistent relaxion of CCSMCs and ED. The persistent flaccidity induces hypoxia of penile tissue. The process will increase the activation of TGF-β pathway and cause penile tissue fibrosis. RhoA/ROCK pathway also participates in penile fibrosis by interacting with TGF-β pathway through S1P molecule. Ligands such as ET-1, Ang II and norepinephrine released from endothelial cells and cavernous nerve terminals bind to the receptors on CCSMCs, corpora cavernosum endothelial cells and fibroblast and activate RhoA/ROCK pathway. The up-regulation of RhoA/ROCK pathway can inhibit the expression and activity of eNOS in endothelial cells. RhoA/ROCK pathway also mediates the contraction of CCSMCs by facilitating the phosphorylation of MLCP and induces the apoptosis of CCSMCs by activating the Akt/Bad/Bax/caspase-3 pathway. The upregulation of Shh pathway also accelerates the apoptosis of CCSMCs. The injury of cavernous nerve and the state of hypoxia cause oxidative stress which produces much free radicals. It also contributes to tissue fibrosis, apoptosis of CCSMCs. These changes of penile tissue also produce oxidative stress conversely. The interaction between these molecules and pathways contributes to the occurrence of ED together.

## 5 Latest therapeutic strategies of cavernous nerve injury-related erectile dysfunction

Cavernous nerve injury causes an imbalance between nitrergic and sympathetic innervation of the penis, followed by long-term hypoxia, oxidative stress, tissue apoptosis and fibrosis (Figure 1). Therefore, therapeutic strategies should focus on cavernous nerve protection and regeneration, improving penile blood supply, reducing oxidative stress and normalizing recognized molecular signaling pathways. At present, exploration of CNI-ED treatments mainly stays in the stage of preclinical trials, but the results are encouraging, and with people paying constant attention to translational medicine, it is believed that there will be more therapeutic methods to enter the clinical research in the future. Emerging therapeutic approaches can be roughly summarized into four categories, namely small molecule and drug, stem cell-based therapy (SCT), micro-energy therapy and platelet-rich plasma (PRP) therapy. Among small molecules, Shh protein, which protects injured nerves and preserves the anatomical integrity of cavernous tissue *via* multiple pathways, has been deeply studied. Another small molecule tyrosine kinase receptor type 1 monoclonal antibody (TrkA-mAb) provides us with an interesting perspective, that is, the utilization of neutralizing antibody to balance the regenerative activity of sympathetic and parasympathetic nerves. Low-intensity extracorporeal shock wave therapy (Li-ESWT) is the first proposed micro-energy therapy for CNI-ED, and its efficacy in vasculogenic ED has received abundant support. Recently, studies of its effect on neurogenic ED are gratifying, indicating that micro-energy therapy will be another considerable choose for neurogenic ED patients. As for PRP, it promotes cavernous nerve regeneration through a variety of growth factors (such as PDGF, VEGF, *etc.*) and cytokines (such as CXCL5) released by platelets (Scott et al., 2019; Wu et al., 2021). There have been several animal studies supporting the therapeutic effect of PRP on CNI-ED to date. Novel and promising therapeutic strategies and their related representative research are specifically introduced in Table 1.

**Table 1:** Novel therapeutic strategies of CNI-Related ED.

Therapeutic strategies	Method and location of administration	Duration of treatment	Curative effect	Potential mechanism	Representative study
Shh protein	Direct delivery into penis or cavernous nerve *via* peptide amphiphile (PA) nanofiber hydrogels vehicle	9 days (penis); 6 weeks (CN)	Improving erectile function of CNI rats	suppressing both caspase 9 and 8 apoptotic mechanisms; promoting CN regeneration	Martin S, 2021 Martin et al. (2021); Angeloni NL, 2011 Angeloni et al. (2011)
Icariside II flavonoid derivative	Dissolved in PEG 400 and administered intragastrically	3 weeks	Improving erectile function of CNI rats	Activating the proliferation and differentiation of penile endogenous stem cells (SCs) via up-regulation of Wnt/β-catenin signaling pathway	Gu SJ, 2021 Gu et al. (2021)
Combination of JNK inhibitor and LIMK2 inhibitor	Intraperitoneal injection	5 weeks	Restoring the cavernous veno-occlusive function (CVOF)	Inhibiting apoptosis and fibrosis of corpus cavernosum to restore CVOF	Cho MC, 2021 Cho et al. (2021)
fidgetin-like 2(FL2)-siRNA	Direct injection into MPG and CN immediately after CNI *via* nanoparticle encapsulation	4 weeks	Improving erectile function of CNI rats	Inhibiting the FL2-mediated severing of dynamic microtubules in distal axon shaft and growth cone to promote CN regeneration	Baker L, 2021 Baker et al. (2021)
proNGF neutralizing antibody	Intracavernous injection	2 weeks	Improving erectile function of CNI mice	Regulating the production of neurotrophic and angiogenic factors in penis	Chung DY, 2021 Chung et al. (2021)
PKA agonist colforsin	Intraperitoneal injection	3 days	Improving erectile function of CNI rats	Enhancing bioactivity of nNOS and reducing oxidative stress	Karakus S, 2017 Karakus et al. (2017)
TrkA-mAb	Intraoperative injection into MPG and postoperative intracavernous injection	6 weeks	Improving erectile function and sexual behavior of CNI rats	Suppressing sympathetic nerve regeneration and facilitating parasympathetic nerve regeneration through blockade of NGF/TrkA pathway	Lin G, 2015 Lin et al. (2015)
Rho-Kinase Inhibitor fasudil	Oral administration	4 weeks	Improving erectile function and CVOF of CNI rats	Inhibiting Akt/Bad/Bax/caspase-3 and LIMK2/cofilin pathways to reduce cavernous apoptosis and fibrosis	Cho MC, 2015 Cho et al. (2015)
VEGF and BDNF combined gene therapy	Transfected rat adipose stem cells (ADSCs) with lentivirus, and then transplanted ADSCs around MPG	2 weeks	Improving erectile function of CNI rats	Collaboratively promoting CN regeneration	Yang W, 2020 Yang et al. (2020)
Induced pluripotent stem cell–derived mesenchymal stem cells (iMSC)	Intracavernous injection	4 weeks	Improving erectile function of CNI rats	Promoting angiogenesis, neurogenesis, anti-apoptosis and anti-oxidative stress *via* paracrine factors such as VEGF, IGF1, NGF	Chen Z, 2019 Chen et al. (2019)
MSC-derived exosomes	Intracavernous injection	4 weeks	Improving erectile function of CNI rats	Inhibiting apoptosis of CCSMCs	Ouyang X, 2018 Ouyang et al. (2018)
LI-ESWT	placed on the suprapubic region and oriented towards the penis	4 weeks	Improving erectile function of pelvic neurovascular injury rats	Preserving neuronal and vascular integrity via induction of VEGF-release and anti-apoptosis	Wang HS, 2019 Wang et al. (2019)
LI-ESWT (in human)	applied to the root of penis, the shaft, and at a few millimeters proximal to the glans	6 weeks	Improving patients’ IIEF-5 scores at 1 month and 1 year after treatment	Same as above	Frey A, 2016 Frey et al. (2016)
Optimized PRP	Intracavernous injection	4 weeks	Improving erectile function of CNI rats	Promoting CN regeneration through a variety of growth factors (such as PDGF, VEGF, etc.) released by platelets	Wu YN, 2016 Wu et al. (2016)

## 6 Future perspectives

Each of these therapeutic categories has advantages and drawbacks, and additional optimization is required to obtain the ultimate clinical application. In terms of small molecules and drugs, oral and intravenous administrations offer the benefits of convenience and high acceptability, however, since medicine is dispersed throughout the body, a higher dose is inevitable to attain the effective concentration locally, necessitating more toxicity and efficacy tests. ICI and intraoperative para-cavernous nerve injection are now more broadly utilized and can successfully enhance local drug concentrations while reducing adverse effects. It is worth noting, though, that there is considerable blood flowing through the corpus cavernosum, and traditionally, blood flow is temporarily restricted *via* ligation of the penile base to lengthen the drug’s action period, but the benefit is limited. Therefore, a sustained release drug delivery system (SRDDS) should be developed in the future, which will substantially minimize the number of ICIs as well as adverse effects. Similarly, a SRDDS is also required for para-CN injection, as it can only be conducted once during surgery. As for SCT, accumulating animal experiments have proven its therapeutic effect on CNI-ED, and several human ED studies have not reported serious complications (Raheem et al., 2021). Nevertheless, its long-term risk and optimal dosing remain unknown, coupled with the difficulty of obtaining autologous stem cells, low expansion ability and high cost limit its application. With the advancement of exosome and iMSC research, and existing studies which suggested that stem cells exert their effects primarily *via* paracrine mechanisms, iMSC-derived exosomes may overcome the limitations mentioned above and are expected to be widely applied in the clinic in the future. In terms of micro-energy therapy, it includes Li-ESWT, low-intensity pulsed ultrasound (LIPUS) and micro-energy acoustic pulse (MAP). The therapeutic effect of Li-ESWT on CNI-ED is well-documented, coupled with its non-invasive and easy-to-perform features, it is likely to be first approved for clinical usage. Efforts should be made in the future to clarify its potential mechanism, optimize the treatment protocol, and monitor the delayed adverse effects that possibly exist. LIPUS and MAP have recently been proven to promote CN regeneration, thus it is hypothesized that they can improve postoperative erectile function, but further evidence is needed (Chiang and Yang, 2019; Peng et al., 2020). PRP is relatively easy to prepare and has shown no clear side effects in limited clinical studies. However, placebo-controlled, multicenter studies that can confirm the efficacy of PRP in CNI-ED remain to be conducted, and the optimal injection dosage and duration of treatment have yet to be determined (Epifanova et al., 2020). In summary, the treatment methods for CNI-ED will be more diversified in the future, and the strategies of combination therapy may further benefit ED patients after RP.

## 7 Conclusion

With deepening research on the pathogenesis of CNI-ED, multiple molecules in the penis and their related pathways have been proven to be involved. Together, these pathways eventually lead to the irreversible damage to the penile structure after cavernous nerve injury, coupled with the wide correlations and interactions of these pathways, which makes the interpretation of the molecular mechanism of CNI-ED more complex. At present, in addition to the progress of molecular signals in the penis, encouraging results have also been achieved in the research on neurological pathways and treatments for nerve regeneration. The combined early rehabilitation strategies of promoting upstream nerve regeneration and recovering abnormal molecular signals of the downstream penis are presumable to save patients’ erectile function after RP. In future studies, the cross-talk among these molecular pathways needs to be further clarified, and the questions of how denervation injury induces the molecular alterations in the penis need to be addressed as well. Despite the fact that there are still numerous issues and obstacles in this field, recent findings indicate that the future is bright and patients are expected to no longer suffer from ED after RP.
